# Event and Comment

**Published:** 1917-03

**Authors:** 


### DENTAL REGISTER

Vol. LXXI MARCH, 1917 No. 3

### EVENT AND COMMENT

THE PRICE OF AN ARTIFICIAL DENTURE. We recently read a statement that the cost of a full denture made by the average dentist was no more than that of a pair of ready-made pants. It started a train of thought as to the comparative values of pants and plates. In estimating real values we may say that one pays ten dollars for a pair of pants and he rarely gets over six months of service out of them; but we have taught our patients that a set of teeth ought to last them the remainder of their lives, or at least for ten or fifteen years.

It may be possible that it costs no more to make a vulcanite denture than it does to make a good pair of pants, so far as actual cost of material and expenditure of time and effort are concerned. Then why should we charge more for the denture than for the pants? As a mere matter of economics perhaps we should not, at least so long as the denture is constructed on the mere mechanical and commercial basis. As a matter of fact there are differences in the manner in which pants are constructed. The average tailor will make to measure a pair of pants for ten dollars, while another tailor will put five dollars more of skill in designing and fitting so as to command the higher fee for satisfaction in service. Curiously enough there are people so particular in this respect that they willingly go to the fifteen-dollar pants maker in preference to the ten-dollar tailor, or to the ready-to-wear clothing shop where he may buy the same goods at a less price. As a rule people do not pay the higher prices because they happen to be able to do so, but they seem to have ideals that can only be satisfied by the highest type of service at their command.

When we ask the better class of dentists why they don't make vulcanite plates they invariably reply that they can't afford to make them for the customary fees. We can't help asking another pertinent question of these same dentists and it is, have you ever qualified to construct ideal dentures, and made any special effort to educate your patients as to the relative merits of a properly designed and constructed denture in contrast with the ordinary ten-dollar plate? We are morally certain that no professional dentist can compete in price with the advertiser, or commercial laboratory, but we are firmly convinced that the people need better prosthetic service today more than ever before, and they are being referred by the better skilled professional dentists to the cheapest available establishment for this most important service. Isn't it high time that the root canal problem be given a short vacation for better assimilation, and that a little attention be given to the subject of artificial dentures made by the dentist, rather than for him by the office boy, or the commercial laboratory? There never was a time when the best possible dentures were in such demand as now, and there never were so many excellent technical methods ready to be applied by the skilled professional dentist as there are today. The materials and technique are provided, but the spirit of high endeavor in this line seems to be woefully lacking. So far as we can see the lack of interest in this important branch of dental service seems to be due to the fact that this service has for so long been rendered on a fixed price basis that it offers no attraction to the skillful dentist. No one seems brave enough to make an effort to overcome this traditionally fixed price and to place this service on the professional-fee basis, as is almost every other service rendered in a modern dental office. Let's stop making plates at so much *per* and give our patients the highest type of service known to the profession for a dignified professional fee.

